# Detecting DNA Modifications from SMRT Sequencing Data by Modeling Sequence Context Dependence of Polymerase Kinetic

**DOI:** 10.1371/journal.pcbi.1002935

**Published:** 2013-03-14

**Authors:** Zhixing Feng, Gang Fang, Jonas Korlach, Tyson Clark, Khai Luong, Xuegong Zhang, Wing Wong, Eric Schadt

**Affiliations:** 1 Tsinghua National Laboratory for Information Science and Technology, and Department of Automation, Tsinghua University, Beijing, China; 2 Department of Statistics, Stanford University, Stanford, California, United States of America; 3 Department of Computer Science and Engineering, University of Minnesota, Minneapolis, Minnesota, United States of America; 4 Pacific Biosciences, Menlo Park, California, United States of America; 5 Department of Genetics and Genomics Sciences, Mount Sinai School of Medicine, New York, New York, United States of America; University of California Davis, United States of America

## Abstract

DNA modifications such as methylation and DNA damage can play critical regulatory roles in biological systems. Single molecule, real time (SMRT) sequencing technology generates DNA sequences as well as DNA polymerase kinetic information that can be used for the direct detection of DNA modifications. We demonstrate that local sequence context has a strong impact on DNA polymerase kinetics in the neighborhood of the incorporation site during the DNA synthesis reaction, allowing for the possibility of estimating the expected kinetic rate of the enzyme at the incorporation site using kinetic rate information collected from existing SMRT sequencing data (historical data) covering the same local sequence contexts of interest. We develop an Empirical Bayesian hierarchical model for incorporating historical data. Our results show that the model could greatly increase DNA modification detection accuracy, and reduce requirement of control data coverage. For some DNA modifications that have a strong signal, a control sample is not even needed by using historical data as alternative to control. Thus, sequencing costs can be greatly reduced by using the model. We implemented the model in a R package named seqPatch, which is available at https://github.com/zhixingfeng/seqPatch.

## Author Summary

DNA modifications have been found in a wide range of living organisms, from bacteria to human. Many existing studies have shown that they play important roles in development, disease, bacteria virulence, etc. However, for many types of DNA modification, for example N6-methyladenine and 8-oxoG, there is not an efficient and accurate detection method. Single molecule real time (SMRT) sequencing not only generates DNA sequences, but also generates DNA polymerase kinetic information. The kinetic information is sensitive to DNA modifications in the sequenced DNA template, and therefore can be used for detecting a wide range of DNA modification types. The usual detection strategy is a case-control method, which compare kinetic information between native sample and a control sample whose modifications have been removed. However, generating a control sample doubles the cost. We proposed a hierarchical model, which can incorporate existing SMRT sequencing data to increase detection accuracy and reduce coverage requirement of control sample or even avoid the need of a control sample in some cases. We tested our method on SMRT sequencing data of plasmids with known modified sites and *E. coli* K-12 strain to demonstrate our method can greatly increase detection accuracy and reduce sequencing cost.

## Introduction

Modifications to individual bases like 5-methylcytosine, 5-hydroxymethylcytosine, and N6-methyladenine in DNA sequences are an important epigenetic component to the regulation of living systems, from individual genes to cellular function. Single molecule, real time (SMRT) sequencing provides a high throughput platform for direct DNA modification detection without the need for special sample preparation procedures such as bisulphite treatment or restriction enzyme digestion [1]–[3]. In SMRT sequencing, each base identity is read when fluorescently labeled nucleotides are incorporated into a DNA sequence being synthesized by DNA polymerase [4]. In this case, because the incorporation events are being directly observed in real time, the duration between the pulses of light (referred to as inter-pulse duration or IPD) that indicate an incorporation event can be precisely measured. IPD measures are a direct reflection of the DNA polymerase kinetics. This kinetic parameter for the enzyme has been shown to be sensitive to a wide range of DNA modification events, including 5-methylcytosine, 5-hydroxymethylcytosine, and N6-methyladenocine [1]–[3], where variations in the kinetics are predictive of modification events.

**Figure 1:**
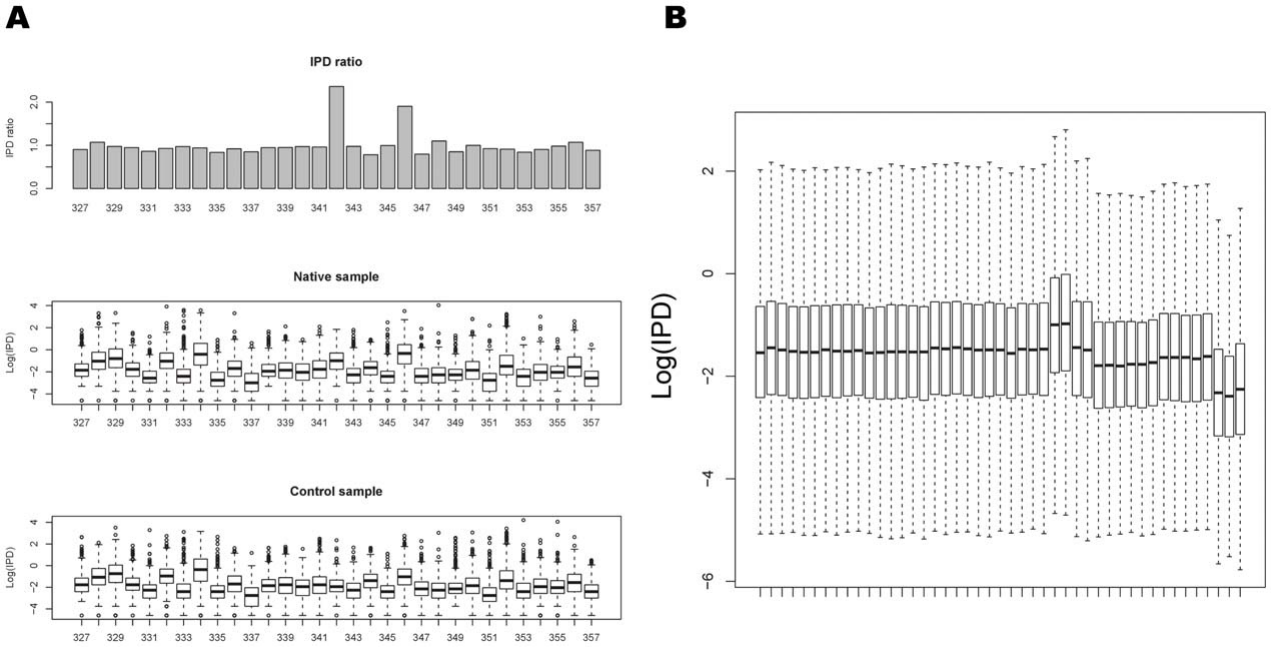
Movie effects and an IPD profile example. (A) IPD distributions of positions 327–357 in the native and control samples (Plasmid m4C native and Plasmid m4C control in Table 1). The middle and bottom panels show IPD distributions(Boxplot) of the native and control sample respectively. The top panel shows ratio of the average IPD between native sample and control sample. Position 342 is modified, and its ratio of the average IPD values between native sample and control sample is much higher than the other positions. In addition, a single modified nucleotide can affect IPD distributions at several flanking bases. Here, the average IPD of position 346 in the native sample is higher than that of the control sample because of the modification event at position 342. (B) Boxplots of IPD distributions of 45 movies of *E. coli* WGA-FCR in Table 1 show that the IPD distribution can undergo large overall shifts across different movies.

For each position in the DNA sequence being synthesized, the IPD distribution is empirically determined as each read covering a given position yields an IPD value for that position, so that for each position there are a number of IPD observations. In these previous demonstrations [1], [2], kinetic variations were detected using a case-control method in which the IPDs at a given site in the native DNA from a sample of interest (case group) are compared to the IPDs in whole-genome amplified (WGA) DNA corresponding to the native DNA (control group). The WGA process erases all of the modifications by replacing any modified base with the corresponding standard base. The null IPD distribution can be determined from the IPDs in the control group and then the IPD distribution for the case group can be compared to this null distribution (Figure 1A). If the IPD values between cases and controls differ significantly, then a kinetic variation event is called. Because SMRT sequencing reads are strand specific with respect to the detection of these kinetic variation events, modifications can be inferred in a strand specific manner. This approach to detecting kinetic variation events works well when there is sufficiently high numbers of reads covering each position, but is much less reliable in low coverage cases due to the high variability of IPD measures (the IPDs are exponentially distributed). In addition, this case-control method requires sequencing a sample twice, so making these detections come at a significant cost.

In this paper, we examine the correlation between polymerase kinetics and sequence context to demonstrate that polymerase kinetics can be well predicted by local sequence context, suggesting that baseline kinetics can be established for any sequence context to use as a null distribution in testing for base modification events. We demonstrate that this correlation between local sequence context and enzyme kinetics is highly consistent across independent experiments carried out on DNA from different species. Given this, we hypothesized that IPDs from positions with the same sequence context, referred to as homologous positions, including those from historical control data, could be used jointly to better estimate the null IPD distribution. Towards that end, we develop a hierarchical model to combine IPDs across homologous positions to enhance the detection of kinetic variation events. The hierarchical model can work with or without control data. When control data are available, for a given position, the hierarchical model combines IPDs of control data and IPDs of homologous positions to estimate the null IPD distribution. We refer to this type of model as a hierarchical model with control data. When there is no control data available, for a given position, the hierarchical model estimates the null IPD distribution using only IPDs of homologous positions from historical data. We refer to this as a hierarchical model without control data. We test these two hierarchical models on two high coverage plasmid datasets and a medium coverage *E. coli* K-12 MG 1655 dataset: 1) plasmid DNA isolated from a strain of *E. coli* engineered to methylate the 4th carbon in cytosine residues, referred to as 4-mC, in the GATC context; 2) plasmid DNA isolated from a strain of *E. coli* engineered to methylate the A residue in the GATC context, referred to as 6-mA; and 3) DNA isolated from a wild type *E. coli* reference strain (K-12) (Table 1). We show that the hierarchical model with control data significantly increases the detection accuracy compared with the case-control design on all of the datasets. The hierarchical model without control data also achieves a good accuracy for N6-methyladenocine, which has a strong signal-to-noise ratio (i.e. impact on the enzyme kinetics), but does not work well for methylcytosine, whose signal-to-noise ratio is relatively weak. In the case of the *E. coli* K-12 dataset, we were able to detect roughly 80% of the 6-mA events in the GATC context at a 5% FDR (False Discovery Rate) using the hierarchical model with control data, a context known to be methylated in a vast majority of the occurrences of the GATC motif in this strain [5]. In addition to detecting these known methyladenine events in the GATC context, we demonstrate the detection of thousands of kinetic variation events that occur at positions not previously described as having known methylation motifs, suggesting more extensive patterns of modification than had been previously observed.

**Table 1:** Samples.

Sample names	Genome size	Coverage per strand	Chemistry	NCBI SRA ID
Plasmid m4C native	3,589 nt	752x	FCR	SRX209633
Plasmid m4C control	3,589 nt	1557x	FCR	SRX209634
Plasmid m6A native	3,591 nt	186x	FCR	SRX188834
Plasmid m6A control	3,591 nt	1486x	FCR	SRX188835
*M. pneumoniae* WGA-FCR	816,394 nt	15x	FCR	SRX209646
*E. coli* WGA-FCR	4,639,675 nt	8x	FCR	SRX209658
*E. coli* native	4,639,675 nt	12x	C2	SRX209659
*E. coli* WGA-N	4,639,675 nt	12x	C2	SRX209660
*E. coli* WGA-C	4,639,675 nt	13x	C2	SRX209661
*M. pneumoniae* WGA-C2	816,394 nt	40x	C2	SRX209662

## Results

### Box-Cox transformation and normalization

**Figure 2:**
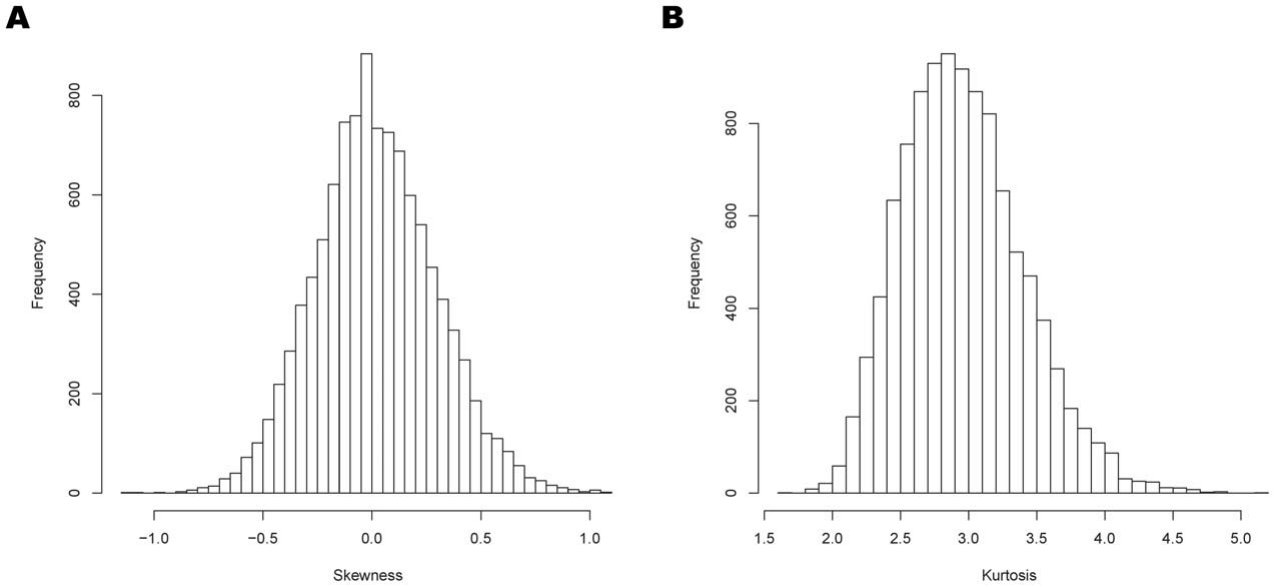
Skewness and kurtosis distribution. For each position, the skewness and kurtosis of its Box-Cox transformed IPDs are plotted. (A) The distribution of skewness of all positions is approximately centered at 0. (B) The distribution of kurtosis is approximately centered at 3.

We note that while the IPDs are observed to be exponentially distributed, tests based on this assumption are more sensitive to extreme outliers. Thus, we adopt a Box-Cox transformation to make the IPDs follow an approximate normal distribution (Figure 2), making it more robust to outliers. Formally, we used the following transformation,
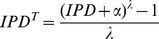
Because the chemistry of SMRT sequencing is being constantly improved, there are two different types of chemistry, FCR and C2, represented in our datasets. The IPD characteristics of these different chemistries are quite different, so we used different 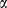 and 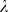, which were estimated for each set. For data using the FCR chemistry, we used 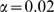 and 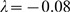. For data using the C2 chemistry, we used 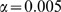 and 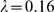. The 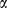 and 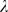 parameter values were chosen empirically such that the skewness distribution was approximately centered at 0.

A number of factors can influence enzyme kinetics in addition to sequence context (see below) and DNA modification, including reagent lot, temperature, SMRTcell lot and instrument operator. Just as we observe batch effects and other experimental noise factors with other technologies such as microarrays and RNA-seq that impact gene expression values, so these different effects can have strong effects on the IPD. Therefore, IPDs from different experiments are not necessarily directly comparable. For the current version of the Pacific Biosciences RS DNA sequencing instrument, DNA molecules are sequenced in zero mode waveguides (ZMWs) located on a SMRTcell [6], with pulses of light in different color channels corresponding to the bases being incorporated into the sequence being synthesized. These signals are detected and recorded by a CCD camera operating at 100 Hz, resulting in a movie containing up to 150,000 pulse streams corresponding to the different ZMWs on the SMRTcell. Overall, the IPD distribution can be significantly different between movies even for identical DNA samples (Figure 1B). Therefore, we applied a simple centering approach to normalize the IPD data before modification detection.
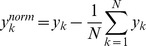
where 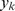 is any Box-Cox transformed IPD in a movie, and N is the number of alignable bases in that movie. In the rest of the paper, we refer to the normalized IPD as simply the IPD.

### Dependence of polymerase kinetic on sequence context

**Figure 3:**
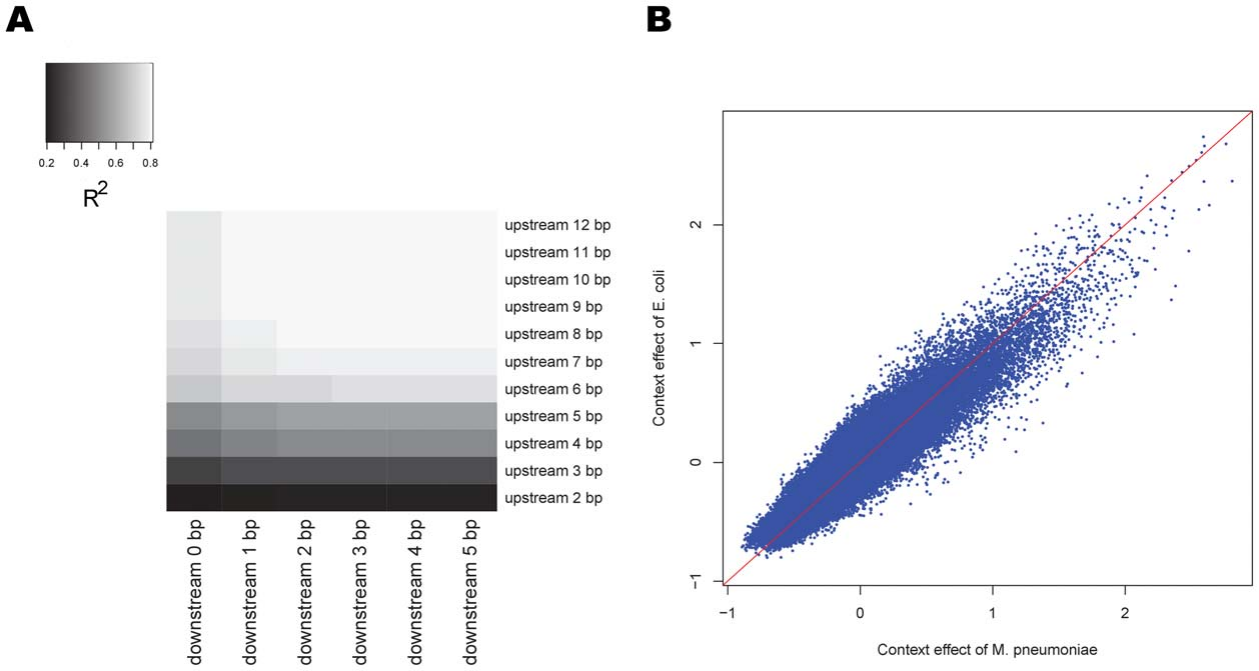
Impact of sequence context on position-specific kinetic rates. (A) Heatmap of 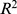 for the position-specific kinetic rate variance explained by sequence context suggests that 7 bases upstream and 2 bases downstream, [−7,+2], of the incorporation site are the most informative. Bases beyond this region do not provide much information about polymerase kinetics. (B) Scatter plot of the [−7,+2] context effect in whole genome amplified *E. coli* and *M. pneumoniae* (*E. coli* WGA-C and *M. pneumoniae* WGA-C2 in the Table 1) shows that context effects are highly consistent between these experiments, with a Pearson's correlation coefficient is 0.91.

The kinetic rate of DNA polymerase is known to be sensitive to sequence context [7]. Given the ability of SMRT sequencing to observe many thousands of individual molecules of DNA polymerase as they carry out DNA synthesis, we examined the relationship between the kinetic rate (estimated from the IPDs) and sequence context. For each position, the position-specific kinetic rate is defined as the mean of its Box-Cox transformed IPDs (Methods). Sequence context is defined as the sequence flanking the incorporation site of interest, the boundaries of which are explored below. To avoid ambiguity caused by modification events, we explored enzyme kinetics using whole-genome amplified (WGA) *E. coli* K-12 data (*E. coli* WGA-FCR in Table 1), given the WGA process erases all chemical modifications. From the K-12 dataset, we extracted positions in which the single strand coverage was greater than 35 reads. We then applied MART [8], a non-linear tree based regression method, to estimate the relationships between polymerase kinetics and sequence context. Here, position-specific kinetic rate is the response variable and sequence context is the predictor variable. The proportion of the variation in the response variable that can be explained by the predictor variable (i.e., the 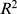 value), was used as the measure of dependence of enzyme kinetics on sequence context. We explored these relationships over different sequence context lengths and found that 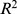 grows as the number of bases upstream of the incorporation site increases, but becomes saturated at 7 bases upstream. The bases downstream from the incorporation site have much smaller impact on the enzyme kinetic rate, with positions more than 2 bases downstream from the incorporation site having no observable impact on the 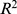 values (Figure 3A). Roughly 80% of the IPD variation can be explained by a 10 base pair sequence context (7 bases upstream and 2 bases downstream from the incorporation site).

We refer to the average Box-Cox transformed IPDs corresponding to positions with the same sequence context as the context effect. We examined the consistency of the context effect between two independent experiments: 1) WGA data from the *E. coli* K-12 strain, and 2) WGA data from *M. pneumoniae* (*E. coli* WGA-C and *M. pneumoniae* WGA-C2 in Table 1). While these experiments were performed completely independently, carried out by two different groups at two geographically separated sites, the context effects were strikingly similar (Figure 3B), with 80% of the IPD variation in one set explained by variation in the second set. Importantly, we compared context effects between two experiments using the same chemistry (FCR chemistry), as the consistency of context effects will not hold when comparing experiments using different chemistries. Thus, in all the experiments carried out herein, only datasets with the same chemistry are used together.

### Detecting DNA modifications by incorporating sequence context information

**Figure 4:**
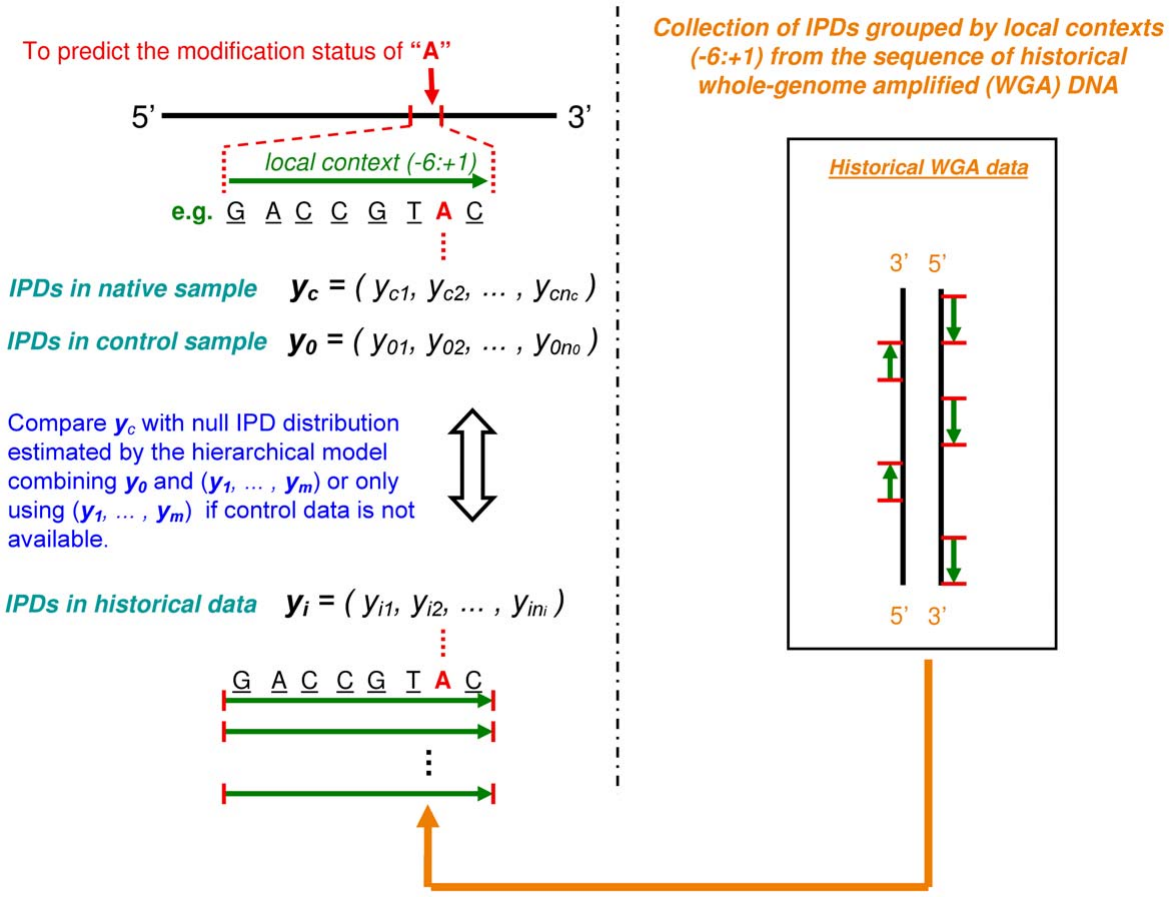
Schematic diagram of the hierarchical model. The red letter A represents the test base, with each read covering this position contributing an IPD, denoted by 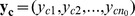 and 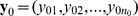 for native and control samples(if available), respectively, where 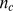 and 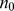 indicate the sequence coverage. IPDs of homologous positions in historical data are denoted by 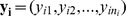, where 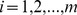 and 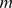 is the number of homologous positions.

Given that a large percentage of variability of position specific enzyme kinetic rates can be explained by sequence context (Figure 3A), IPDs of homologous positions can be combined together to estimate the null IPD distribution. In addition, because of the high consistency in context effects across different experiments, IPDs of homologous positions in historical WGA datasets can also be incorporated to enhance the power to detect kinetic variation events (Figure 4). However, the IPD distributions of homologous positions will not be exactly the same, and so, false positive calls may be introduced if the null IPD distribution is estimated without considering the heterogeneity in the IPD distributions that can exist between homologous positions. To deal with this type of heterogeneity, we developed an hierarchical model to incorporate IPDs of homologous positions in a robust fashion. In the hierarchical model, Box-Cox transformed IPDs of homologous positions were assumed to follow normal distributions, with differences in mean and variance allowed between these distributions. The mean and variance parameters were treated as random variables and were assumed to follow the same prior distribution. For the hierarchical model with control data, the model was fitted by both IPDs from the control data and as well as IPDs from all homologous positions. For the hierarchical model without control data, the model was fitted using only homologous positions in historical data. Then, we adopt a likelihood ratio to evaluate how likely a position is modified (See Methods).

**Figure 5:**
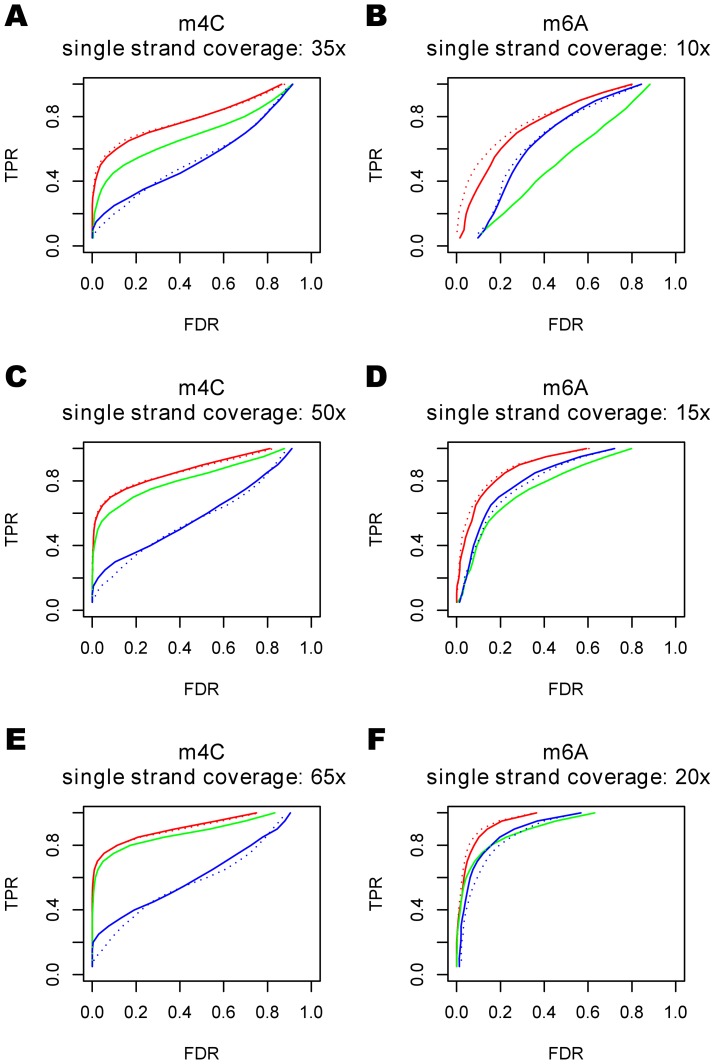
Performance of the hierarchical model in plasmid data. The red, green and blue curves are ROC curves for the hierarchical model with control data, the case-control method, and the hierarchical model without control data, respectively. These three methods were tested on two different datasets: 1) a 3,589 bases long plasmid with 19 known 4-methylcytosines(4-mC) under single strand coverage 35x,50x,and 65x, respectively(A,C,E), and 2) a 3,591 bases long plasmid with 23 known N6-methyladenines(6-mA) under single strand coverage 15x,20x,25x(B,D,F). The solid lines are ROC curves using a [−6,+1] sequence context and the dotted lines are ROC curves using the [−7,+2] sequence context.

To assess the utility of the hierarchical model in detecting kinetic variation events, we compared the naive case-control design with the hierarchical model using data from two data sets in which the sites that were modified were known a priori. The first set was generated from plasmid DNA isolated from a strain of *E. coli* engineered to methylate the 4th carbon of cytosine residues in each GATC context (referred to as the 4-mC set), and the second was generated from plasmid DNA isolated from a strain of *E. coli* engineered to methylate adenine residues in each GATC context (referred to as the 6-mA set). We use *E. coli* WGA-FCR and *M. pneumoniae* WGA-FCR in Table 1 as historical data and only contexts that have more than 5 positions with larger than 10x coverage in the historical data are used. We explored both the [−7,+2] contexts (7 bp upstream, 2 bp downstream of the incorporation site) and [−6,+1] contexts, and found that their performances were similar (Figure 5). However, roughly one third of the positions in these datasets did not have the corresponding [−7,+2] context in the historical data. Therefore, to make the comparisons fair, we only considered positions that had a corresponding context in the historical data. To maximize the number of sequence contexts in one dataset that would be represented in another, we restricted the sequence context to 8 bases(the [−6,+1] context) for the remainder of our study. For the hierarchial model with control data (see Methods), when the sequence coverage of the control sample is relatively low (15x
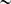
35x single strand coverage), the hierarchical model compared to the case-control method is seen to increase the sensitivity by 10%
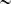
30% under the same FDR (Figure 5). Performance of the case-control method and hierarchical model become similar as sequencing coverage of the control sample increases. For the hierarchical model without control (See Methods), the accuracy is comparable to the case-control method for 6-mA, but does not perform as good for 4-mC.

We further tested our method on datasets with partial modifications in which only a fraction of the molecules with respect to a given position were modified. Given one of the limitations with the modeling approach presented herein is that it assumes in the native sample a given position is either fully modified or not, understanding the sensitivity of this assumption on the detection rates is important. For each of the 6-mA set and 4-mC datasets, we constructed an artificial native sample, where only a certain proportion of reads were sampled from the native sample (referred to as the modification proportion), while others were sampled from the control data. We tested the performance of our method on three different modification proportions: 50%, 70% and 90%. As expected, the detection accuracy decreases as the modification proportion decreases, however, in all cases our method was still able to make detections even when the fully modified assumption was clearly violated. We further note that it is possible to achieve accuracy that is comparable to the case of fully modified positions by increasing the sequence coverage of the native sample (Figure S1).

### Detecting DNA modifications in *E. coli* K-12

To further evaluate the performance of the hierarchical model, we explored data from the *E. coli* K-12 strain in which there are not only modifications that are known to occur in certain sequence motifs (e.g. GATC), but also potentially novel modification events that cannot be explained by known motifs. We applied both hierarchical modeling with and without control data to the SMRT sequencing data from native the *E. coli* K-12 MG 1655 strain (*E. coli* native in Table 1). For the hierarchical model with control data, we used data from a WGA *E. coli* K-12 MG 1655 sample as control (*E. coli* WGA-C in Table 1) and a WGA *M. pneumoniae* data (*M. pneumoniae* WGA-C2 in Table 1), which is generated in another unrelated experiment, as historical data. For each given position, the IPD distribution in the native sample was compared to the null IPD distribution, which was estimated by fitting a hierarchical model that combines the IPDs of the corresponding positions in the control sample and IPDs of all of the homologous positions. Homologous positions were identified in two different ways: 1) find homologous positions in the WGA *M. pneumoniae* data, and 2) find homologous positions in the control data. For the hierarchical model without control, we estimated the null distribution by fitting the hierarchical model by the homologous positions in the WGA *M. pneumoniae* data only. A position was called modified if the generated likelihood ratio exceeded a certain threshold (see Methods).

**Figure 6:**
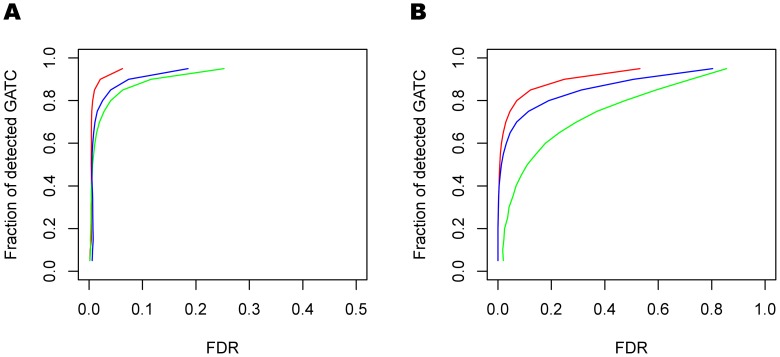
Performance of the hierarchical model in *E. coli* K-12 data. The red, green and blue curves are ROC curves for the hierarchical model with control data, the case-control method, and the hierarchical model without control data, respectively. (A) shows the ROC curve when the detection region is restricted to 20 bp around the adenine residue in the GATC context, and (B) shows the ROC curve for the whole genome detection.

As most adenines in the GATC context are expected to be methylated in wild type *E. coli* K-12 MG 1655, we detected modifications in the regions within 20 bp around adenine positions in the GATC context to evaluate how well 6-mA could be detected. Here, the FDR is estimated as the ratio between the number of significant adenines detected that are not in the GATC context and the total number of significant adenines detected. We note that it is certainly possible that there are modified bases outside of the GATC context, so that treating only adenines detected in the GATC context as true positives and all other bases as true negatives, out estimated FDR can be considered as a conservative estimation, i.e. the actual FDR is lower than this. The receiver operating characteristic (ROC) curve (Figure 6A) shows that 95% of adenine of GATC can be detected under FDR of 5% by using the hierarchical model with control. The hierarchical model greatly increases the detection accuracy in this instance compared to the case-control method. We can also detect 6-mA without the control data, where the accuracy is lower than hierarchical model with control, but the results are comparable to the naive case-control method. If we apply this detection approach on genome-wide scale, we detect many putative modification events, with the ROC curve (Figure 6B) showing that at the 5% FDR we not only detect 80% of the adenine in the GATC context by using hierarchical model with control, but we also identify about 2000 other positions in other contexts that may reflect off target activity of the methyltransferase that makes the 6-mA modifications in the GATC context or perhaps reflects the activity of other enzymes capable of inducing base modifications. In the genome-wide study, to estimate FDR, we detected DNA modifications in another WGA sample (*E. coli* WGA-N in Table 1), where no modification should be found, and FDR is estimated by ratio between number of DNA modifications detected in the WGA sample(*E. coli* WGA-N in Table 1) and number of modifications detected in the native sample (*E. coli* native in Table 1).

## Discussion

We examined the correlation between DNA polymerase kinetics and sequence context quantitatively and found that roughly 80% of the variation in the enzyme kinetics as measured by IPD variance can be explained by sequence context. Our data support that the most informative regions of sequence context for the enzyme kinetics at a given incorporation site is the region 7 bp upstream and 2 bp downstream of the incorporation site. In addition, we found that this context dependence is extremely consistent between independent SMRT sequencing experiments carried out using the same chemistry. IPDs of homologous positions, including those from historical control data can therefore be incorporated to improve DNA modification detection accuracy. However, because heterogeneity of the IPD distribution within the same sequence context can cause false positive events, we adopted a hierarchical model that can adaptively incorporate information from homologous positions. The hierarchical model is flexible in that it can be used with or without control data. We demonstrated that the hierarchical model with control data can greatly increase accuracy compared to the naive case-control method. For the types of modifications that have a relatively weak signal-to-noise ratio, such as 4-mC, the hierarchical model without control does not perform as good as the case-control method. This may be expected given the sequence context in such instances does not appear to explain all of the kinetic variation, with other factors such as fragment length and experimental condition perhaps dominating the estimation of the null distribution from historical data. However, for modification types with a strong signal-to-noise ratio, noisy null IPD distributions have a relatively small impact on accuracy.

Our results suggest that the hierarchical model can reduce the requirement of control samples and thus provide a significant cost benefit. For detecting modifications with a strong signal-to-noise ratio, one can generate low coverage control data or even avoid the generation of the control data altogether. It may be possible in the future as more SMRT sequencing data obtains, given the dependence of local sequence context on enzyme kinetics, to build null models specific to each sequence context to leverage as a control in detecting base modification events. We anticipate as well that as more sequence data obtains across different species with larger genomes than the prokaryotic genomes represented in our study, that we will be able to re-evaluate whether a more expanded sequence context around the incorporation site better explains the DNA polymerase enzyme kinetics. It may be that with an expanded set that considers 9 bases upstream of the incorporation site and 3 bases downstream, for example, a better explanation of the enzyme kinetics obtains. Further, as the historical datasets get larger, we may also find that the historical data on its own achieves the same results as the combined historical and control data in all contexts and for all modification types.

## Methods

### Characterizing the relationship between sequence context and polymerase kinetics

For the 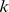
th position in the genome, we assume that its Box-Cox transformed IPDs follow a normal distribution, which is
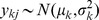
where 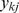 is the jth Box-Cox transformed IPD of the 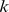
th position in the genome, and 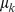 is the position specific polymerase kinetic rate, which is by its sequence context. We used 
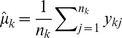
 (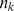 is the single strand coverage of the 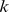
th position), which are the estimated position specific polymerase kinetic rates, as the response, and the corresponding sequence context as the predictor to build a non-linear regression model using the MART method [8]. The dataset we employed for this characterization is whole genome amplified *E. coli* K-12 data after outlier removal and coverage filtering. Each data point in the dataset is a pair of estimated position specific polymerase kinetic rates and sequence contexts, which represent the upstream and downstream bases of the position of interest. Performance of the regression approach was evaluated using 5-fold cross validation in which 80% of the data points were randomly selected as the training set, MART was trained on this set, and then the predicted responses were carried out for the remaining 20% of the data set. The 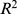 was the statistic used to measure the performance and was calculated as 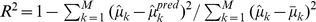, where 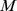 is the sample size, 
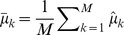
, and 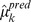 is the predicted 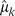.

### Hierarchical model with control data

#### Alternative model

For a given position, the Box-Cox transformed IPDs in the native and control samples were 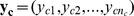 and 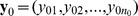, respectively, where 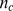 and 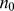 represent the sequence coverage of each. Box-Cox transformed IPDs of the homologous positions are denoted by 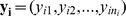, i = 1,…,m, where 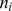 is the sequence coverage and m is the number of homologous positions. We assume that the Box-Cox transformed IPDs follow a normal distribution.
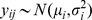
where 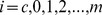 and 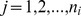. We assume that 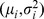, 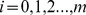, come from the same conjugate prior distribution, i.e.
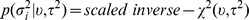


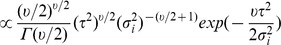


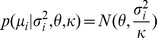


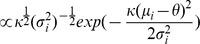
where 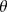, 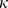, 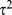 and 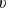 are hyperparameters, and 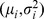, 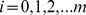, are independent and identically distributed given hyperparameters. In addition, we also assume the variance of the Box-Cox transformed IPDs in the native sample, i.e. 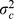 is generated by the same prior distribution with 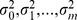, is
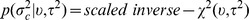
The means of the IPDs in the native sample, i.e. 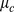, is treated as hyperparameters rather than being assumed to have the same prior distribution with 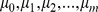, given putative DNA modification events may have an impact on 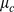 and thus make it deviate from that prior distribution. Therefore, given the hyperparameters, the marginal log-likelihood function is
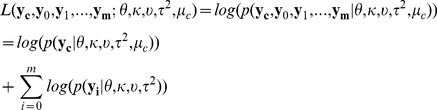
(1)By integrating out 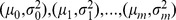 in 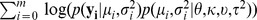, we get
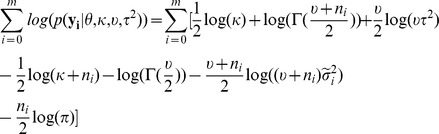
where i = 0,1,…,m, and 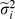 is the scale parameter of the posterior distribution of 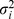, which is [9]

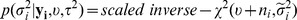
where 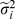 is
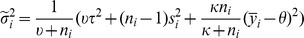
where 
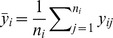
 and 
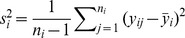
.

By integrating out 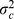 in 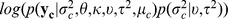, we get
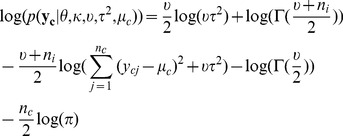
(2)It is obvious that given 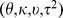, formula (2) can be maximized by setting 
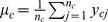
, which is denoted by 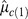. By substituting 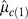 into the formula (1), 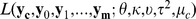 can be maximized by Algorithm 1 in the Text S1. The solution is denoted by 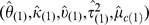.

To evaluate how likely a base is to be modified, we use the marginal log-likelihood ratio, which is
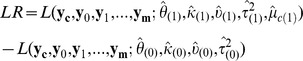
where 
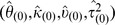
 are hyperparameters maximizing the marginal log-likelihood function, 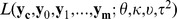, when the null hypothesis, 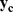 and 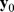 having the same distribution, is true(null model). A base is called modified if 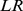 exceeds a certain threshold.

#### Null model

By assuming the null hypothesis is true, we pool 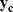 and 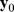 together and denote the pooled sample as 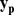. We assume
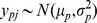
where 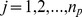, and 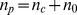. 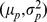 are assumed to have the same prior distribution with 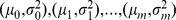, which is
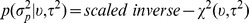


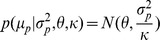
By integrating out 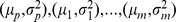 in 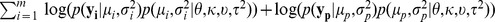, we can get
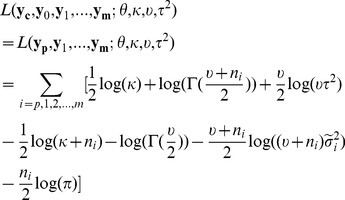
(3)Thus, 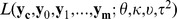 can be maximized by Algorithm 1 described in the Text S1, and the solution is denoted as 
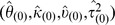


### Hierarchical model without control data

The hierarchical model without control data is a special case of the hierarchical model with control data, i.e. 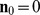 or vector 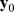 is empty.

#### Alternative model

By simply removing 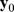 in (1), we can get the hierarchical model without control data, and the marginal log-likelihood is
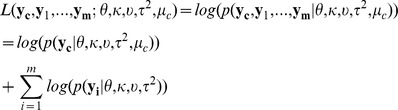
The 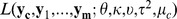 can be maximized by Algorithm 1 in the Text S1, and the solution is 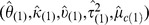.

#### Null model

By assuming the null model is true, i.e.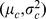 are generated by the same prior distribution with 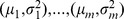, the likelihood function 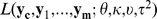 could be got by setting 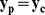 in (3). Thus, we use
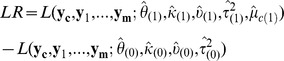
to evaluate how likely the base is modified. where 
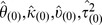
 is the parameter maximizing likelihood 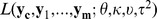 when null model is true.

### Evaluation of DNA modification detection methods

We evaluated case-control method and hierarchical model on two different datasets: 3589 bases long plasmid with 19 known 4-methylcytosines, and 3591 bases long plasmid with 23 known N6-methyladenines (Plasmid m4C native/control and Plasmid m6A native/control in Table 1). Whole genome amplified *E. coli* and *M. pneumoniae* data were used as historical data (*E. coli* WGA-FCR and *M. pneumoniae* WGA-FCR in Table 1). A detection is called correct only if its distance to the nearest true modified position is less than or equal to 5 bp. Different thresholds of 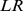 were set, and the corresponding false discovery rate and true positive rate were calculated. False discovery rate and true positive rate are defined as
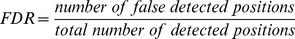


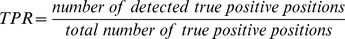
respectively. To get the ROC under different coverage, we randomly sampled reads without replacement 100 times to get average FDR under different TPRs.

### Data access

The raw sequence data listed in Table 1 are available at http://www.ncbi.nlm.nih.gov/sra, under accession number SRA062773 and SRA058893. (SRA058893 was published in [10]).

## Supporting Information

Figure S1 **Performance of the hierarchical model in partially modified plasmid data.** The red, green and blue curves are ROC curves for the hierarchical model with control data, the case-control method, and the hierarchical model without control data, respectively. These three methods were tested on two different datasets: 1) a 3,589 bases long plasmid with 19 known 4-methylcytosines(4-mC) where 50%, 70%, and 90% molecules from the modified site are actually modified on average (single strand coverage of native sample and control sample are 200x and 65x, respectively)(A,C,E), and 2) a 3,591 bases long plasmid with 23 known N6-methyladenines(6-mA) where 50%, 70%, and 90% molecules from the modified site are actually modified on average (single strand coverage of native sample and control sample are 100x and 20x, respectively) (B,D,F).
(TIFF)

Text S1 **EM algorithm for fitting the hierarchial model.** Text S1 provides a detailed description of the EM (Expectation-Maximization) algorithm used for estimating hyperparameters of the proposed hierarchical model.
(PDF)
