# Supplementary figures and images for: Detecting DNA Modifications from SMRT Sequencing Data by Modeling Sequence Context Dependence of Polymerase Kinetic
Source: PLoS Comput Biol. 2013 Mar 14;9(3):e1002935. doi: 10.1371/journal.pcbi.1002935 (PMC3597545; doi:10.1371/journal.pcbi.1002935)

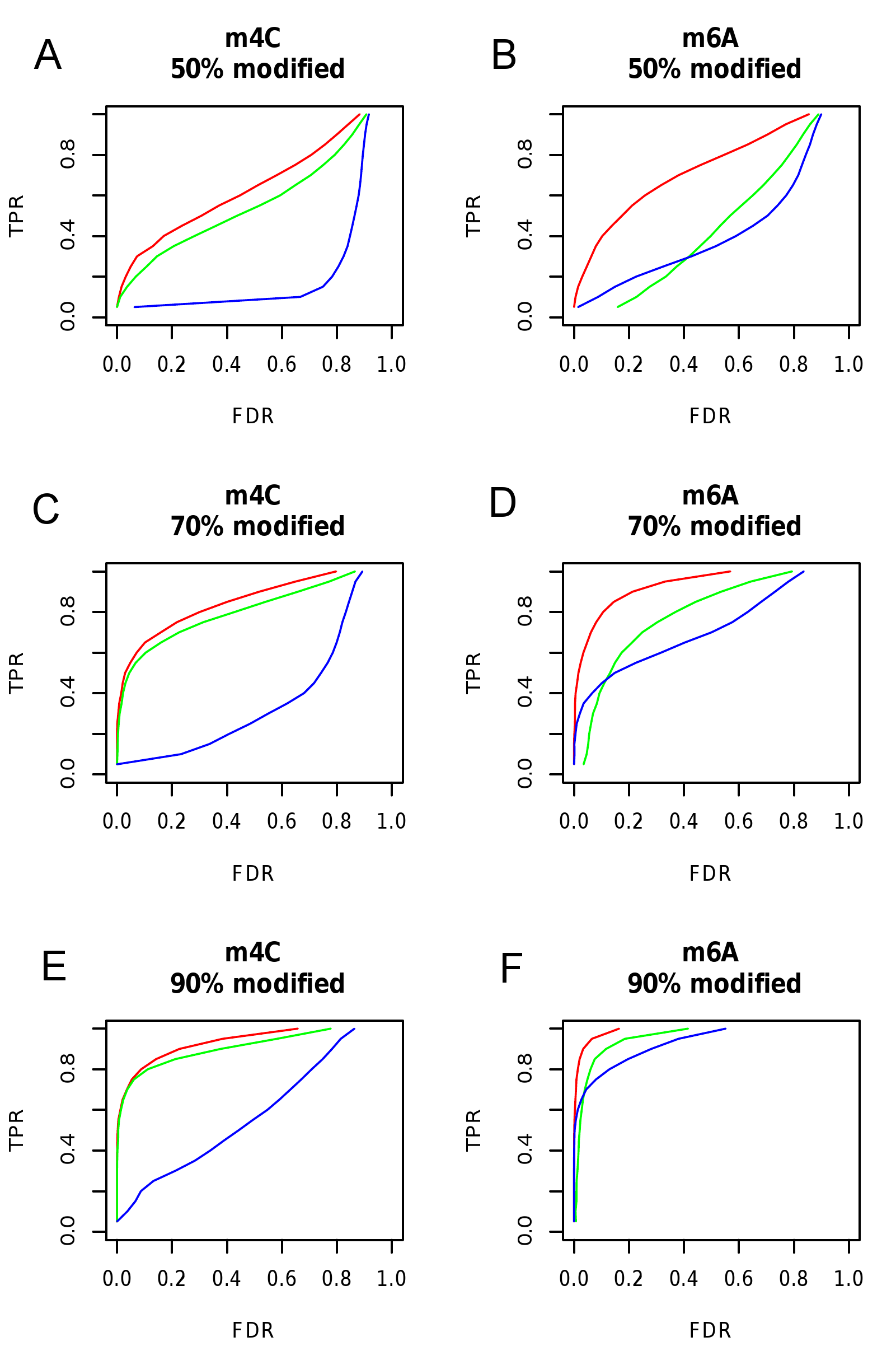

Supplement: Figure S1 — Performance of the hierarchical model in partially modified plasmid data. The red, green and blue curves are ROC curves for the hierarchical model with control data, the case-control method, and the hierarchical model without control data, respectively. These three methods were tested on two different datasets: 1) a 3,589 bases long plasmid with 19 known 4-methylcytosines(4-mC) where 50%, 70%, and 90% molecules from the modified site are actually modified on average (single strand coverage of native sample and control sample are 200x and 65x, respectively)(A,C,E), and 2) a 3,591 bases long plasmid with 23 known N6-methyladenines(6-mA) where 50%, 70%, and 90% molecules from the modified site are actually modified on average (single strand coverage of native sample and control sample are 100x and 20x, respectively) (B,D,F). (TIFF) [file pcbi.1002935.s001.tiff]
