# Supplementary material for: Detecting DNA Modifications from SMRT Sequencing Data by Modeling Sequence Context Dependence of Polymerase Kinetic
Source: PLoS Comput Biol. 2013 Mar 14;9(3):e1002935. doi: 10.1371/journal.pcbi.1002935 (PMC3597545; doi:10.1371/journal.pcbi.1002935)
Supplement: Text S1 — EM algorithm for fitting the hierarchial model. Text S1 provides a detailed description of the EM (Expectation-Maximization) algorithm used for estimating hyperparameters of the proposed hierarchical model. (PDF) [file pcbi.1002935.s002.pdf]

Detecting DNA modifications from SMRT sequencing data by modeling sequence context dependence of polymerase kinetic

Zhixing Feng[1,4], Gang Fang[2], Jonas Korlach[3], Tyson Clark[3], Khai Luong[3], Xuegong Zhang[1], Wing Wong[4,*], and Eric Schadt[3,5,*]

[1]Tsinghua National Laboratory for Information Science and Technology, and Department of Automation, Tsinghua University, Beijing 100084, China; [2]Department of Computer Science and Engineering, University of Minnesota, Minneapolis, MN 55455; [3]Pacific Biosciences, 1380 Willow Road, Menlo Park, CA 94025; [4]Department of Statistics, Stanford University, Stanford, CA 94305; [5]Department of Genetics and Genomics Sciences, Mount Sinai School of Medicine, New York, NY 10029; [*]Correspondence: whwong@stanford.edu, eric.schadt@mssm.edu

Text S1

Hyperparameters estimation for alternative model

In this section, we explain how to estimate hyperparameters by assuming control sample is available. As hierarchical model without control data is basically a special case of hierarchical model with control data, one can simply remove y_0, μ_0 and σ_0^2 to get hyperparameter estimation when control sample is not available, and algorithm is unchanged. When alternative model is true (see section **Hierarchical model with control data** and **Hierarchical model without control data** in the main text), posterior distribution of μ_i and σ_i^2, $i = 0, 1, ..., m$, are[1]

$$p(\mu_i|\mathbf{y_i}, \sigma_i^2, \theta, \kappa) = N(\frac{\kappa}{\kappa + n_i}\theta + \frac{n_i}{\kappa + n_i}\overline{y}_i \ , \ \frac{\sigma_i^2}{\kappa + n_i})$$

$$p(\sigma_i^2|\mathbf{y_i}, \upsilon, \tau^2) = scaled\ inverse - \chi^2(\upsilon + n_i, \widetilde{\sigma}_i^2)$$

where

$$\widetilde{\sigma}_i^2 = \frac{1}{\upsilon + n_i}\Big(\upsilon\tau^2 + (n_i - 1)s_i^2 + \frac{\kappa n_i}{\kappa + n_i}(\overline{y}_i - \theta)^2\Big) \tag{1}$$

$\bar{y}_i = \frac{1}{n_i}\sum_{j=1}^{n_i} y_{ij}$ and $s_i = \frac{1}{n_i-1}\sum_{j=1}^{n_i}(y_{ij} - \bar{y}_i)^2$. Posterior distribution of σ_c^2 is[1]

$$p(\sigma_c^2|\mathbf{y_c}, \upsilon, \tau^2) = scaled\ inverse - \chi^2(\upsilon + n_c, \widetilde{\sigma}_c^2)$$

where

$$\widetilde{\sigma}_c^2 = \frac{1}{\upsilon + n_c}\Big(\upsilon\tau^2 + (n_i - 1)s_c^2\Big)$$

We used posterior expectations of μ_i and σ_i^2 as their estimations, which are

$$\hat{\mu_i} \quad = \quad E(\mu_i|\mathbf{y_i}, \theta, \kappa) = \frac{\kappa}{\kappa + n_i}\theta + \frac{n_i}{\kappa + n_i}\overline{y}_i \qquad (2)$$

where $i = 0, 1, ..., m$.

$$\hat{\sigma_i^2} = E(\sigma_i^2|\mathbf{y_i}, \upsilon, \tau^2) = \frac{n_i + \upsilon}{n_i + \upsilon - 2}\widetilde{\sigma}_i^2$$

where $i = c, 0, 1, ..., m$. We estimate hyperparameters $(\theta, \kappa, \upsilon, \tau^2, \mu_c)$ from the data by maximizing the marginal log-likelihood function, which is

$$L(\mathbf{y_c}, \mathbf{y_0}, \mathbf{y_1}, ..., \mathbf{y_m}; \theta, \kappa, \upsilon, \tau^2, \mu_c) = log(p(\mathbf{y_c}, \mathbf{y_0}, \mathbf{y_1}, ..., \mathbf{y_m}|\theta, \kappa, \upsilon, \tau^2, \mu_c))$$

$$= log(p(\mathbf{y_c}|\theta, \kappa, \upsilon, \tau^2, \mu_c))$$

$$+ \sum_{i=0}^{m} log(p(\mathbf{y_i}|\theta, \kappa, \upsilon, \tau^2))$$

where

$$\log(p(\mathbf{y_c}|\theta, \kappa, \upsilon, \tau^2, \mu_c)) = \frac{\upsilon}{2}\log(\upsilon\tau^2) + \log(\Gamma(\frac{\upsilon + n_i}{2}))$$

$$- \frac{\upsilon + n_i}{2}\log(\sum_{j=1}^{n_c}(y_{cj} - \mu_c)^2 + \upsilon\tau^2) - \log(\Gamma(\frac{\upsilon}{2}))$$

$$- \frac{n_c}{2}\log(\pi)$$

$$\sum_{i=0}^{m} log(p(\mathbf{y_i}|\theta, \kappa, \upsilon, \tau^2)) = \sum_{i=0}^{m}[\frac{1}{2}\log(\kappa) + \log(\Gamma(\frac{\upsilon + n_i}{2})) + \frac{\upsilon}{2}\log(\upsilon\tau^2)$$

$$- \frac{1}{2}\log(\kappa + n_i) - \log(\Gamma(\frac{\upsilon}{2})) - \frac{\upsilon + n_i}{2}\log((\upsilon + n_i)\widetilde{\sigma}_i^2)$$

$$- \frac{n_i}{2}\log(\pi)]$$

It is obvious that $L(\mathbf{y_c}, \mathbf{y_0}, \mathbf{y_1}, ..., \mathbf{y_m}; \theta, \kappa, \upsilon, \tau^2, \mu_c)$ can be maximized by setting $\mu_c = \frac{1}{n_c}\sum_{j=1}^{n_c} y_{cj}$. However, it is difficult to get a close form of $(\theta, \kappa, \upsilon, \tau^2)$ and we therefore adopted a EM algorithm (Algorithm 1) to esti-

mate them numerically.

In the EM procedure, μ_i and σ_i^2 were regarded as missing data, and the log-likelihood function with the complete data was

$$
\begin{aligned}
&l(\mathbf{y}, \mu, \boldsymbol{\sigma^2}; \theta, \kappa, \tau^2, \upsilon) \\
=& \log(p(\mathbf{y}|\mu, \boldsymbol{\sigma^2})) + \log(p(\mu|\boldsymbol{\sigma^2}, \theta, \kappa)) + \log(p(\boldsymbol{\sigma^2}|\upsilon, \tau^2))) \\
=& \log(p(\mathbf{y}|\mu, \boldsymbol{\sigma^2})) + \sum_{i=0}^{m} \log(p(\mu_i|\sigma_i^2, \theta, \kappa)) + \sum_{i=c,0,1,\ldots,m} \log(p(\sigma_i^2|\tau^2, \upsilon)) \quad (3)
\end{aligned}
$$

where $\mathbf{y} = (\mathbf{y_c}, \mathbf{y_0}, \mathbf{y_1}, ..., \mathbf{y_m})$, $\mu = (\mu_0, \mu_1, ..., \mu_m)$ and $\boldsymbol{\sigma^2} = (\sigma_c^2, \sigma_0^2, \sigma_1^2, ..., \sigma_m^2)$. Initial values $(\theta_0, \kappa_0, \tau_0^2, \upsilon_0)$ were assigned to $(\theta, \kappa, \tau^2, \upsilon)$, and in the tth step $(t \geq 1)$ of EM algorithm, $(\theta, \kappa, \tau^2, \upsilon)$ were updated by the optimal value $(\theta_{opt}, \kappa_{opt}, \tau_{opt}^2, \upsilon_{opt})$ maximizing $E\big(l(\mathbf{y}, \mu, \boldsymbol{\sigma^2}; \theta, \kappa, \tau^2, \upsilon) \mid \mathbf{y}, \theta_{t-1}, \kappa_{t-1}, \tau_{t-1}^2, \upsilon_{t-1}\big)$, where E(.) is expectation in term of (μ, σ^2), i.e. $\theta_t = \theta_{opt}, \kappa_t = \kappa_{opt}, \tau_t = \tau_{opt}^2, \upsilon_t = \upsilon_{opt}$. This procedure was repeated until convergence.

In the tth step of the EM algorithm, $(\theta_{opt}, \kappa_{opt})$ and $(\tau_{opt}^2, \upsilon_{opt})$ can be obtained by maximizing posterior expectation of the second term and the third term of equation (3), i.e. $\sum_{i=0}^{m} E\big(\log(p(\mu_i|\sigma_i^2, \theta, \kappa)) \mid \mathbf{y}, \theta_{t-1}, \kappa_{t-1}, \tau_{t-1}^2, \upsilon_{t-1}\big)$ and $\sum_{i=c,0,1,\ldots,m} E\big(\log(p(\sigma_i^2|\tau^2, \upsilon)) \mid \mathbf{y}, \theta_{t-1}, \kappa_{t-1}, \tau_{t-1}^2, \upsilon_{t-1}\big)$, respectively.

[1] $\Delta l = E\big(l(\mathbf{y}, \mu, \boldsymbol{\sigma^2}; \theta_{opt}, \kappa_{opt}, \tau_{opt}^2, \upsilon_{opt}) \mid \mathbf{y}, \theta_{t-1}, \kappa_{t-1}, \tau_{t-1}^2, \upsilon_{t-1}\big) - E\big(l(\mathbf{y}, \mu, \boldsymbol{\sigma^2}; \theta_{t-1}, \kappa_{t-1}, \tau_{t-1}^2, \upsilon_{t-1}) \mid \mathbf{y}, \theta_{t-1}, \kappa_{t-1}, \tau_{t-1}^2, \upsilon_{t-1}\big)$

Algorithm 1 EM algorithm for hyperparameters estimation

Assign initial values to hyperparameters, $\theta = \theta_0$, $\kappa = \kappa_0$, $\upsilon = \upsilon_0$ and $\tau^2 = \tau_0^2$.
Set $t = 1$
while $\Delta l \leq 0.1$ [1] **do**
  E-step: Calculate conditional expectation of log likelihood function in terms of μ_i and σ_i^2, i.e. $E\big(l(\mathbf{y}, \mu, \sigma^{\mathbf{2}}; \theta, \kappa, \tau^2, \upsilon) \mid \mathbf{y}, \theta_{t-1}, \kappa_{t-1}, \tau_{t-1}^2, \upsilon_{t-1}\big)$.
  M-step: Update $(\theta, \kappa, \upsilon, \tau^2)$ by setting $\theta_t = \theta_{opt}, \kappa_t = \kappa_{opt}, \upsilon_t = \upsilon_{opt}, \tau_t = \tau_{opt}^2$, where $(\theta_{opt}, \kappa_{opt}, \upsilon_{opt}, \tau_{opt}^2)$ are hyperparameters maximizing $E\big(l(\mathbf{y}, \mu, \sigma^{\mathbf{2}}; \theta, \kappa, \tau^2, \upsilon) \mid \mathbf{y}, \theta_{t-1}, \kappa_{t-1}, \tau_{t-1}^2, \upsilon_{t-1}\big)$
  Set $t = t + 1$
end while

Estimating θ and κ By taking posterior expectation of the second term of equation (3), we can get

$$\begin{aligned}
&\sum_{i=0}^{m} E\big(\log(p(\mu_i|\sigma_i^2, \theta, \kappa)) \mid \mathbf{y}, \theta_{t-1}, \kappa_{t-1}, \tau_{t-1}^2, \upsilon_{t-1}\big) \\
= \ & -\frac{\kappa}{2}\sum_{i=0}^{m}\big(\frac{1}{\kappa_{t-1}+n_i} + \frac{(\theta - \hat{\mu}_{i(t-1)})^2}{\widetilde{\sigma}_{i(t-1)}^2}\big) - \frac{m+1}{2}\log(\kappa) + C
\end{aligned}$$

where $\widetilde{\sigma}_{i(t-1)}^2$ and $\hat{\mu}_{i(t-1)}$ are estimated $\widetilde{\sigma}_i^2$ and μ_i given $(\theta_{t-1}, \kappa_{t-1}, \tau_{t-1}^2, \upsilon_{t-1})$ (equation (1) and (2)), and C is a constant, which doesn't contain any hyperparameters. $\sum_{i=0}^{m} E\big(\log(p(\mu_i|\sigma_i^2, \theta, \kappa)) \mid \mathbf{y}, \theta_{t-1}, \kappa_{t-1}, \tau_{t-1}^2, \upsilon_{t-1}\big)$ can be

maximized by θ_{opt} and κ_{opt}, which are

$$\begin{aligned}\theta_{opt} &= \sum_{i=0}^{m} \frac{\hat{\mu}_{i(t-1)}}{\widetilde{\sigma}^2_{i(t-1)}} / \sum_{i=0}^{m} \frac{1}{\widetilde{\sigma}^2_{i(t-1)}} \\ \kappa_{opt} &= (m+1) / \sum_{i=0}^{m} \big(\frac{1}{\kappa_{t-1}+n_i} + \frac{(\theta_{opt}-\hat{\mu}_{i(t-1)})^2}{\widetilde{\sigma}^2_{i(t-1)}} \big)\end{aligned}$$

Estimating τ^2 and υ By taking posterior expectation of the third term of equation (3), we can get

$$\begin{aligned}&\sum_{i=c,0,1,\ldots,m} E\big(\log(p(\sigma_i^2|\tau^2,\upsilon)) \mid \mathbf{y}, \theta_{t-1}, \kappa_{t-1}, \tau^2_{t-1}, \upsilon_{t-1} \big) \\ =\; & \frac{(m+2)\upsilon}{2} \log(\frac{\upsilon\tau^2}{2}) - (\frac{\upsilon}{2}+1) \sum_{i=c,0,1,\ldots,m} E\big(\log(\sigma_i^2) \mid \mathbf{y}, \theta_{t-1}, \kappa_{t-1}, \tau^2_{t-1}, \upsilon_{t-1} \big) - \\ & \frac{\tau^2\upsilon}{2} \sum_{i=c,0,1,\ldots,m} E\big(\frac{1}{\sigma_i^2} \mid \mathbf{y}, \theta_{t-1}, \kappa_{t-1}, \tau^2_{t-1}, \upsilon_{t-1} \big) - (m+2)\log(\Gamma(\frac{\upsilon}{2})) \\ =\; & \frac{(m+2)\upsilon}{2} \log(\frac{\upsilon\tau^2}{2}) - (\frac{\upsilon}{2}+1) \sum_{i=c,0,1,\ldots,m} (\log(\frac{(\upsilon_{t-1}+n_i)\widetilde{\sigma}^2_{i(t-1)}}{2}) - \psi(\frac{\upsilon_{t-1}+n_i}{2})) - \\ & \frac{\tau^2\upsilon}{2} \sum_{i=c,0,1,\ldots,m} \frac{1}{\widetilde{\sigma}^2_{i(t-1)}} - (m+2)\log(\Gamma(\frac{\upsilon}{2}))\end{aligned}$$

where $\Gamma(.)$ is gamma function and $\psi(.)$ is digamma function.

By setting

$$\begin{cases} \frac{\partial}{\partial \tau^2} \sum_{i=c,0,1,...,m} E\big(\log(p(\sigma_i^2|\tau^2, \upsilon)) \mid \mathbf{y}, \theta_{t-1}, \kappa_{t-1}, \tau_{t-1}^2, \upsilon_{t-1}\big) = 0 \\ \frac{\partial}{\partial \frac{\upsilon}{2}} \sum_{i=c,0,1,...,m} E\big(\log(p(\sigma_i^2|\tau^2, \upsilon)) \mid \mathbf{y}, \theta_{t-1}, \kappa_{t-1}, \tau_{t-1}^2, \upsilon_{t-1}\big) = 0 \end{cases}$$

we got

$$\begin{cases} \frac{(m+2)\upsilon}{2\tau^2} - \frac{\upsilon}{2} \sum_{i=c,0,1,...,m} \frac{1}{\tilde{\sigma}_{i(t-1)}^2} = 0 \\ (m+2)\big(\log(\frac{\upsilon}{2}) + \log(\tau^2) - \psi(\frac{\upsilon}{2})\big) - \sum_{i=c,0,1,...,m} (\log(\frac{(\upsilon_{t-1}+n_i)\tilde{\sigma}_{i(t-1)}^2}{2}) - \psi(\frac{\upsilon_{t-1}+n_i}{2})) = 0 \end{cases}$$

we got close form solution of the above equations by using approximation of digamma function, which is $\psi(\frac{\upsilon}{2}) \approx \log(\frac{\upsilon}{2}) - \frac{1}{\upsilon} - \frac{1}{3\upsilon^2}$.

$$\begin{aligned} \tau_{opt}^2 &= \frac{m+2}{\sum_{i=c,0,1,...,m} \frac{1}{\tilde{\sigma}_{i(t-1)}^2}} \\ \upsilon_{opt} &= \frac{2}{3(\sqrt{1+\frac{4}{3}T} - 1)} \end{aligned}$$

where $T = \frac{1}{m+2} \sum_{i=c,0,1,...,m} \big(\log(\frac{(\upsilon_{t-1}+n_i)\tilde{\sigma}_{i(t-1)}^2}{2}) - \psi(\frac{\upsilon_{t-1}+n_i}{2})\big) - \log(\tau_{opt}^2)$.

Hyperparameters estimation for null model

For hierarchical model with control data, we denote pooled Box-Cox transformed IPD of native and control data as $\mathbf{y_p}$ (i.e. $(y_{c1}, y_{c2}, ..., y_{cn_c}, y_{01}, y_{02}, ..., y_{0n_0})$). For hierarchical model without control data, we simply let $\mathbf{y_c}$, Box-Cox transformed IPD of native sample, equal to $\mathbf{y_p}$, because it is a special case of

hierarchical model with control data, in which y_0 is empty. We assume $\mathbf{y_p}$ follows a normal distribution

$$\mathbf{y_p} \sim N(\mu_p, \sigma_p^2)$$

and $(\mu_p, \sigma_p^2), (\mu_1, \sigma_1^2), (\mu_2, \sigma_2^2), ..., (\mu_m, \sigma_m^2)$ have the same prior distribution, which is

$$p(\sigma_i^2|\upsilon, \tau^2) = scaled\ inverse - \chi^2(\upsilon, \tau^2)$$
$$p(\mu_i|\sigma_i^2, \theta, \kappa) = N(\theta, \frac{\sigma_i^2}{\kappa})$$

where, $i = p, 1, ..., m$. posterior distribution of μ_i and σ_i^2, $i = p, 1, ..., m$, are

$$p(\mu_i|\mathbf{y_i}, \sigma_i^2, \theta, \kappa) = N(\frac{\kappa}{\kappa + n_i}\theta + \frac{n_i}{\kappa + n_i}\overline{y}_i\ ,\ \frac{\sigma_i^2}{\kappa + n_i})$$
$$p(\sigma_i^2|\mathbf{y_i}, \upsilon, \tau^2) = scaled\ inverse - \chi^2(\upsilon + n_i, \widetilde{\sigma}_i^2)$$

where

$$\widetilde{\sigma}_i^2 = \frac{1}{\upsilon + n_i}\Big(\upsilon\tau^2 + (n_i - 1)s_i^2 + \frac{\kappa n_i}{\kappa + n_i}(\overline{y}_i - \theta)^2\Big)$$

[1]. We used posterior expectations of μ_i and σ_i^2 as their estimations, which are

$$\hat{\mu_i} = E(\mu_i|\mathbf{y_i}, \theta, \kappa) = \frac{\kappa}{\kappa + n_i}\theta + \frac{n_i}{\kappa + n_i}\overline{y}_i$$
$$\hat{\sigma_i^2} = E(\sigma_i^2|\mathbf{y_i}, \upsilon, \tau^2) = \frac{n_i + \upsilon}{n_i + \upsilon - 2}\widetilde{\sigma}_i^2$$

where $i = p, 1, ..., m$. Like the previous section, we adopt a EM algorithm (Algorithm 1) to maximize marginal log-likelihood function $L(\mathbf{y_p}, \mathbf{y_1}, ..., \mathbf{y_m}; \theta, \kappa, \upsilon, \tau^2)$. In the EM procedure, μ_i and σ_i^2 were regarded as missing data, and the log-likelihood function with the complete data was

$$\begin{aligned} & l(\mathbf{y}, \mu, \boldsymbol{\sigma^2}; \theta, \kappa, \tau^2, \upsilon) \\ = & \log(p(\mathbf{y}|\mu, \boldsymbol{\sigma^2})) + \sum_{i=p,1,...,m} \log(p(\mu_i|\sigma_i^2, \theta, \kappa)) \\ + & \sum_{i=p,1,...,m} \log(p(\sigma_i^2|\tau^2, \upsilon)) \end{aligned} \tag{4}$$

where $\mathbf{y} = (\mathbf{y_p}, \mathbf{y_1}, ..., \mathbf{y_m})$, $\mu = (\mu_p, \mu_1, ..., \mu_m)$ and $\boldsymbol{\sigma^2} = (\sigma_p^2, \sigma_1^2, ..., \sigma_m^2)$.

Estimating θ and κ By taking posterior expectation of the second term of equation (4), we can get

$$
\begin{aligned}
&\sum_{i=p,1,\ldots,m} E\big(\log(p(\mu_i|\sigma_i^2,\theta,\kappa)) \mid \mathbf{y},\theta_{t-1},\kappa_{t-1},\tau_{t-1}^2,\upsilon_{t-1}\big) \\
= \;& -\frac{\kappa}{2}\sum_{i=p,1,\ldots,m}\big(\frac{1}{\kappa_{t-1}+n_i}+\frac{(\theta-\hat{\mu}_{i(t-1)})^2}{\widetilde{\sigma}_{i(t-1)}^2}\big)-\frac{m+1}{2}\log(\kappa)+C
\end{aligned}
$$

Thus, $\sum_{i=p,1,\ldots,m} E\big(\log(p(\mu_i|\sigma_i^2,\theta,\kappa)) \mid \mathbf{y},\theta_{t-1},\kappa_{t-1},\tau_{t-1}^2,\upsilon_{t-1}\big)$ can be maximized by

$$
\begin{aligned}
\theta_{opt} &= \sum_{i=p,1,\ldots,m}\frac{\hat{\mu}_{i(t-1)}}{\widetilde{\sigma}_{i(t-1)}^2}\Big/\sum_{i=0}^{m}\frac{1}{\widetilde{\sigma}_{i(t-1)}^2} \\
\kappa_{opt} &= (m+1)\Big/\sum_{i=p,1,\ldots,m}\big(\frac{1}{\kappa_{t-1}+n_i}+\frac{(\theta_{opt}-\hat{\mu}_{i(t-1)})^2}{\widetilde{\sigma}_{i(t-1)}^2}\big)
\end{aligned}
$$

Estimating τ^2 and υ By taking posterior expectation of the third term of equation (4), we can get

$$
\begin{aligned}
&\sum_{i=p,1,\ldots,m} E\big(\log(p(\sigma_i^2|\tau^2,\upsilon)) \mid \mathbf{y},\theta_{t-1},\kappa_{t-1},\tau_{t-1}^2,\upsilon_{t-1}\big) \\
=&\frac{(m+1)\upsilon}{2}\log(\frac{\upsilon\tau^2}{2})-(\frac{\upsilon}{2}+1)\sum_{i=p,1,\ldots,m}(\log(\frac{(\upsilon_{t-1}+n_i)\widetilde{\sigma}_{i(t-1)}^2}{2})-\psi(\frac{\upsilon_{t-1}+n_i}{2}))- \\
&\frac{\tau^2\upsilon}{2}\sum_{i=p,1,\ldots,m}\frac{1}{\widetilde{\sigma}_{i(t-1)}^2}-(m+1)\log(\Gamma(\frac{\upsilon}{2})) \qquad (5)
\end{aligned}
$$

By using approximation of digamma function, which is $\psi(\frac{\upsilon}{2}) \approx \log(\frac{\upsilon}{2}) - \frac{1}{\upsilon} - \frac{1}{3\upsilon^2}$, (5) can be maximized by

$$
\begin{aligned}
\tau^2_{opt} &= \frac{m+1}{\sum_{i=p,1,\ldots,m} \frac{1}{\tilde{\sigma}^2_{i(t-1)}}} \\
\upsilon_{opt} &= \frac{2}{3(\sqrt{1+\frac{4}{3}T}-1)}
\end{aligned}
$$

where $T = \frac{1}{m+1} \sum_{i=p,1,\ldots,m} \big(\log(\frac{(\upsilon_{t-1}+n_i)\tilde{\sigma}^2_{i(t-1)}}{2}) - \psi(\frac{\upsilon_{t-1}+n_i}{2})\big) - \log(\tau^2_{opt})$.

References

[1] Gelman A, Carlin JB, Stern HS, Rubin DB (2003) Bayesian Data Analysis. Chapman and Hall/CRC, 2nd edition.
